# Proteomic Perspectives on KRAS-Driven Cancers and Emerging Therapeutic Approaches

**DOI:** 10.3390/curroncol32110614

**Published:** 2025-11-02

**Authors:** Ramesh Karki, Ru Chen, Sheng Pan

**Affiliations:** 1 The Brown Foundation Institute of Molecular Medicine, University of Texas Health Science Center at Houston, Houston, TX 77030, USA; ramesh.karki@uth.tmc.edu; 2 Section of Gastroenterology and Hepatology, Department of Medicine, Baylor College of Medicine, Houston, TX 77030, USA; 3 Department of Integrative Biology and Pharmacology, McGovern Medical School, University of Texas Health Science Center at Houston, Houston, TX 77030, USA

**Keywords:** KRAS, cancer, proteomics, interactome, post-translational modifications (PTMs), KRAS inhibition

## Abstract

**Simple Summary:**

As one of the most frequently mutated oncogenes, the KRAS protein has long been a target of intense therapeutic interest. In this review, we summarize and discuss the current knowledge of proteomic alterations associated with oncogenic KRAS mutations, with particular emphasis on allele-specific proteome signatures and the roles of post-translational modifications (PTMs) of KRAS in modulating functional networks. Furthermore, we highlight recent therapeutic advances targeting KRAS variants and examine emerging resistance mechanisms from a proteomics-informed perspective.

**Abstract:**

KRAS mutations are implicated in approximately 23% of all human malignancies, with particularly high prevalence in pancreatic ductal adenocarcinoma (PDAC) (~92%), colorectal cancer (CRC) (~49%), and non-small cell lung cancer (NSCLC) (~35%). The recent approval of the KRAS^G12C^-specific inhibitors for NSCLC represents a pivotal advancement in KRAS-targeted therapy. Nevertheless, the emergence of intrinsic and acquired resistance to KRAS-targeted therapies poses a significant clinical obstacle to targeting KRAS, which necessitates a deeper understanding of the resistance mechanisms. Recent progress in proteomic studies has enabled comprehensive profiling of the proteomic alterations driven by KRAS mutations, offering valuable insights into the disrupted KRAS interactome, aberrant signaling pathways and dysregulated cellular processes contributing to tumorigenesis. This review discusses current knowledge on proteomic alterations associated with oncogenic *KRAS* mutations, with particular focus on allele-specific proteome signatures and the roles of post-translational modifications (PTMs) of KRAS in modulating the functional networks. Furthermore, we highlight recent therapeutic advances targeting KRAS variants and discuss emerging resistance mechanisms from a proteomics-informed perspective.

## 1. Introduction

KRAS is one of the most frequently mutated oncoprotein, implicated in approximately 23% of all human cancers. Its highest mutation frequency is observed in pancreatic ductal adenocarcinoma (PDAC), where it is altered in up to 92% of cases, followed by appendiceal adenocarcinoma (61%), small bowel adenocarcinoma (53%), colorectal cancer (49%), and non-small cell lung cancer (NSCLC) (35%) [1]. Given its central role in oncogenesis, KRAS has long been a target of intense therapeutic interest, though earlier efforts to inhibit its activity were largely unsuccessful. However, the recent success of covalent inhibitors targeting the KRAS^G12C^ mutation has reinvigorated the field, prompting a surge in research to develop targeted therapies for other KRAS mutations [2]. It is crucial to investigate how oncogenic KRAS functionally influences the proteomic and genomic landscape, particularly its role in remodeling the tumor microenvironment (TME) and driving cancer progression [3,4].

Due to the often poor to moderate correlation between mRNA expression and protein abundance within cells, genomic analysis alone is not sufficient to elucidate the biological processes governed by proteins in tumors entirely [5,6]. Proteomics now enables the comprehensive quantification of global protein expression, degradation, and a wide range of post-translational modifications (PTMs)—including glycosylation, ubiquitination, SUMOylation, palmitoylation, phosphorylation, and acetylation—offering profound insights into cellular functions and disease mechanisms [7]. Mass spectrometry (MS) plays a central role in proteomics by enabling the sensitive and specific identification and quantification of proteins. It allows in-depth and detailed analysis of proteome profiles, turnover dynamics, and PTMs, which are essential for understanding cellular signaling and functional changes in cellular homeostasis [8]. Additionally, it supports the large-scale characterization of clinical specimens, advancing biomarker discovery and therapeutic development [9,10].

MS-based proteomics has been increasingly applied in precision medicine [11]. Its unique ability of multiplexing capacity, high sensitivity, and specificity make it suitable for clinical studies to investigate various diseases, including cancer [12]. Serum proteomics by MS led to the discovery of the first FDA-approved multiplex biomarker OVA1 for detecting ovarian cancer [13]. Other FDA-approved MS-based proteomics in clinical laboratories include the identification of wild-type (WT) amyloid β (Aβ) to detect Alzheimer’s disease, microbes and AMR profile for detecting infectious disease, and Gram-negative and Gram-positive bacteria and yeast to identify infectious disease [14]. Continuous advancements in automation, the standardization of operating procedures and software, and the integration of machine learning in data interpretation are facilitating the transition of MS from research-focused applications to clinical use [15,16,17].

Determining the mutational status of KRAS is essential before initiating treatment plans for cancers such as non-small cell lung cancer (NSCLC) and colorectal cancer (CRC), particularly when considering therapies targeting the epidermal growth factor receptor (EGFR) [18]. This is because mutant KRAS can activate downstream signaling independently of EGFR, rendering EGFR-targeted treatments ineffective. Although DNA sequencing is widely regarded as the gold standard for detecting *KRAS* mutations, proteomic analysis presents distinct advantages in specific clinical contexts. These include situations where tissue samples are limited, biopsies require decalcification that compromises DNA integrity, or when samples are formalin-fixed and paraffin-embedded (FFPE), which can hinder the effectiveness of DNA sequencing [19,20]. The advancement in targeted data-independent acquisition (DIA) analysis for detecting different RAS mutations from FFPE tumor samples [19] and achieving quantitative target engagement analysis of KRAS^G12C^ with its inhibitor (AZD4625) in the FFPE xenograft model proved that MS is an indispensable tool not only for KRAS testing from FFPE samples but also for measuring drug response from FFPE samples that cannot be analyzed by DNA-sequencing-based methods [20].

By outlining the biological role of KRAS and its oncogenic mutations in cancer development, we review recent findings from proteomic studies, including global profiling, PTM characterization, spatial proteomics, and exosome analyses, in KRAS-mutant cancers, examining how different mutations alter the proteomic landscape in cancer cells and contribute to shaping the TME. Furthermore, we highlight recent advances in targeting mutant KRAS and present emerging insights into associated resistance mechanisms.

## 2. Impact of KRAS Mutations on Cellular Proteome Homeostasis

**Figure 1:**
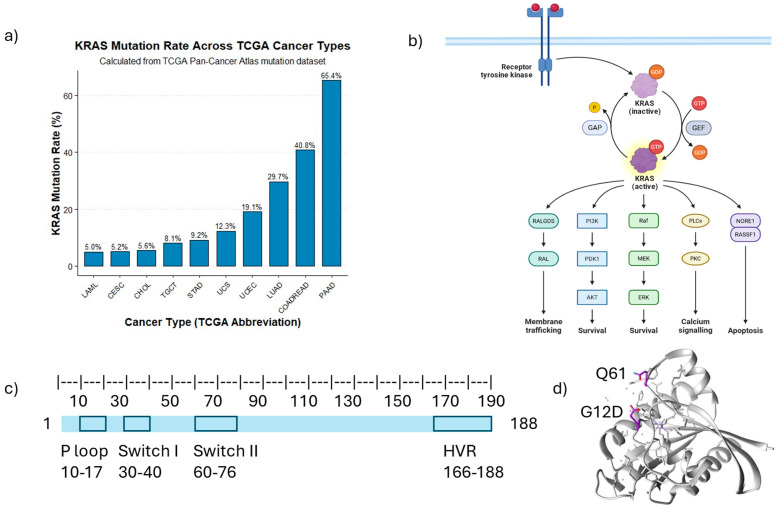
Illustrations of KRAS mutation rates in various cancers and related biological pathways: (**a**) KRAS mutation rate in different cancers generated from data extracted from TGCA PanCancer Atlas downloaded from cBioPortal (www.cbioportal.org). (**b**) Schematic figure showing wild-type KRAS involved in different biological pathways. The diagram was prepared using the BioRender template (https://app.biorender.com/illustrations/6904dffeffa590856ef11875, accessed on 29 October 2025). Different colors represent different pathways that KRAS regulates. (**c**) Schematic representation of the KRAS protein chain with highlighted catalytic regions (Figure generated based on the concept from [2]). (**d**) PDB of G12D mutated KRAS (source: https://www.rcsb.org. PDB ID: 7T47) [22]. Residues G12D and G561 are highlighted in purple. (Abbreviation: PAAD: Pancreatic adenocarcinoma; COADREAD: Colorectal Adenocarcinoma; LUAD: Lung Adenocarcinoma; UCEC: Uterine corpus endometrial carcinoma; UCS: Uterine Carcinosarcoma; STAD: Stomach Adenocarcinoma; TGCT: Testicular Germ Cell Tumors; CHOL: Cholangiocarcinoma; CESC: Cervical Squamous Cell Carcinoma and Endocervical Adenocarcinoma; LAML: Acute Myeloid Leukemia).

KRAS mutations are among the most frequently observed genetic alterations across a wide range of human cancers. Figure 1a illustrates the KRAS mutation rate across different cancers based on data from TGCA PanCancer Atlas. These mutations often lead to the persistent activation of KRAS, resulting in the disruption of normal cellular homeostasis, uncontrolled cell growth, and other major hallmarks of cancer development. Figure 1b shows the biological pathways associated with wild-type (WT) KRAS. Metabolic reprogramming is a well-recognized hallmark of cancer, allowing cancer cells to adapt to their rapid growth and proliferation demands. KRAS mutations lead to the reprogramming of cellular metabolism, leading to distinct metabolic profiles in cancer cells compared to those harboring WT KRAS. A metabolomic analysis of the NSCLC cell line NCI-H1299 and corresponding xenograft models revealed that KRAS^G12C^-overexpressing cells exhibit distinct metabolic reprogramming compared to their WT KRAS counterparts [3,21]. Specifically, KRAS^G12C^ cells showed reduced levels of glutamine and glutamate, alongside elevated concentrations of carnitine, acetylcarnitine, and butyrylcarnitine. These alterations suggest a metabolic shift toward enhanced glutaminolysis and mitochondrial β-oxidation of fatty acids, reflecting an adaptive response to meet the heightened energy demands of rapidly proliferating tumor cells [3,21].

The metabolic reprogramming associated with KRAS mutations is accompanied by a reshaped proteomic landscape that promotes aggressive tumor behavior. Chong et al. analyzed the publicly available proteomic data from the Clinical Proteomic Tumor Analysis Consortium (CPTAC) colon cancer database, and demonstrated that tumors with WT KRAS exhibit a more active immune microenvironment, characterized by the enrichment of immune-related markers such as CD177, MMP1, and ARG1, suggesting a potentially better responsiveness to immunotherapies [23]. In contrast, KRAS-mutant CRC tumors were enriched with proteins like IGFBP2 and KRT18, indicative of a more aggressive, tumor-promoting molecular phenotype [23]. Further analysis of transcriptomic, proteomic, and phosphoproteomic data from the CPTAC colon cancer cohorts showed significant upregulation of tumor migration markers (TGFBR2-S553 and EPHB3) and PI3K/AKT activation (PIP4K2C and PIK3R1) in KRAS-mutated tumors, while cellular metabolism markers (DGAT1 and HINT3) and immune regulation markers (TAP1/2, IFITM1, and IFIH1-S301) were enriched in the WT KRAS tumors [23].

KRAS mutations also modulate the signaling intensity within tumors. Phosphoproteomic analysis of SW48 CRC cells revealed that KRAS^G12D^ and KRAS^G13D^ mutations led to increased tyrosine phosphorylation, including the activation of MAPK and AKT pathways, compared to WT KRAS cells. This elevated phosphorylation correlates with enhanced proliferative and survival signaling in cancer cells [24]. Moreover, global proteome analysis revealed that, compared to CRC cells with WT KRAS, cells with KRAS^G12D^ or KRAS^G13D^ were enriched in pathways related to the cell cycle, DNA replication, and proteoglycans [24].

Proteomic analysis of exosomes from DKO-1 cells (isogenic colorectal cancer cells) expressing mutant KRAS revealed the enrichment of tumor-promoting proteins in exosomes compared to those from DKs-8 cells that express only WT KRAS. Furthermore, the transfer of exosomes from DKO-1 cells (mutant KRAS allele) to DKs-8 cells (WT KRAS allele) significantly enhanced the size and number of DKs-8 colonies in three-dimensional culture compared to the transfer of DKs-8 exosomes, indicating that mutant *KRAS* not only alters the exosomal proteome but also promotes the growth of exosome recipient cells [25].

Baldelli et al. studied the influence of a co-existing WT KRAS allele alongside the mutant KRAS on signaling networks in lung cancer cells using a reverse-phase protein array (RPRA) and demonstrated that retaining the WT KRAS allele within tumors harboring mutant KRAS can lead to significantly different signaling as compared to in cells that have only the mutant KRAS allele [26]. Cells lacking the WT KRAS allele have significant upregulation of RAS-GRF1 and EGFR as compared to cells harboring both the WT and mutant KRAS alleles [26]. Moreover, cells with the presence of the WT KRAS allele together with mutant *KRAS* were sensitive towards the FoxM1 inhibitor siomycin, suggesting that the targeting of FoxM1 could be a therapeutic approach in the treatment of lung cancer with both the WT and mutant KRAS alleles [26].

Zhou et al.’s comprehensive review emphasized the pivotal role of WT KRAS in influencing both tumor progression and regression in cancers harboring mutant KRAS, highlighting its context-dependent effects on disease trajectory and therapeutic response [27]. Using RNA sequencing data, Yan et al. showed that the presence of the WT KRAS allele plays a tumor suppressor role in PDAC cell lines by upregulating the HIPPO pathway, which downregulates the activity of YAP1, a transcriptional co-activator in cell proliferation and survival [28]. Proteomic analysis comparing the protein expression profiles between the cytoplasm and the nucleus showed that cells expressing WT KRAS had higher retention of YAP1 in the cytoplasm as compared to the nucleus, suggesting decreased oncogenic activities of YAP1, reducing tumorigenesis [28]. In contrast, using immunoprecipitation, Matallanas et al. showed that the presence of the WT KRAS allele increases the oncogenic activity of the mutant KRAS allele by inhibiting the mutant KRAS-induced MST2-LATS1 apoptotic pathway. Together with the involvement of WT KRAS, mutant KRAS activates EGFR and inhibits the MST2 pathway through AKT activation [29].

Collectively, KRAS mutations profoundly reshape the proteomic, metabolic, signaling, and TME landscapes of cancer cells, driving tumor aggressiveness, altering immune responses, and influencing therapeutic outcomes—highlighting the need for mutation-specific and context-aware strategies in KRAS-targeted cancer treatment. Table 1 summarizes the proteomic alterations associated with oncogenic KRAS mutations in different biological processes of the cells.

## 3. Distinct KRAS Mutations Associated with Various Functional Changes

KRAS mutations exhibit distinct tissue-specific patterns. The most common mutations in KRAS in PDAC include G12D, G12V, G12R, and Q61H. These mutations take place in the substrate binding site of KRAS, which includes the P-loop and switch II region (Figure 1c,d). In CRC, G12D, G12V, and G13D mutations are predominant, whereas lung cancer frequently harbors G12C, G12V, and G12D mutations [45]. To investigate the functional specificity of KRAS mutations (G12V, G12D, or G13D) in CRC, particularly the variability between the codon 13 and codon 12 KRAS mutants, Hammond et al. employed a SILAC-based quantitative approach to analyze the proteome and phosphoproteome of isogenic KRAS mutant SW48 CRC cell lines. Their findings revealed that codon 12 and codon 13 mutations in KRAS drive distinct signaling pathways through differential protein expression and phosphorylation, contributing to mutation-specific cancer progression and proliferation [46]. Gene Ontology (GO) analysis showed that the codon 12 mutation activates pathways associated with cell adhesion, cytoskeletal organization, and proliferation. In contrast, codon 13 mutant cells exhibited a reduced expression of mitochondrial proteins. Notably, G12D and G12V mutations led to the increased expression and phosphorylation of key proteins such as MET, Caveolin-1, and DCLK1, compared to G13D. Conversely, relative to parental lines, G13D-mutant cells upregulated protein ZO-2 and ALDH3A1, which were downregulated in codon 12 mutant cells. The elevated DCLK1 expression in codon 12 mutant cells was shown to be transcriptionally regulated by KRAS, as RNA interference targeting KRAS reduced DCLK1 protein levels by over 50% [46].

In another study, Tahir et al. explored differential tyrosine kinase signaling in KRAS^G12D^ and KRAS^G13D^ mutants using SILAC-based quantitative proteomics to assess global protein expression and phosphotyrosine (pTyr) levels. G12D mutations were associated with upregulation of proteins involved in glycolysis, fatty acid metabolism, and mitochondrial function, particularly those linked to oxidoreductase activity. In contrast, G13D mutations enhanced the expression of cell surface markers, cytokines, kinases, and transcription factors [24]. Phosphotyrosine profiling revealed that G12D mutations led to the increased phosphorylation of kinases such as MET, MAPK3, MINK, and MPZL1, a protein that activates the tyrosine phosphatase PTPN11. Additionally, proteins involved in focal adhesion and adherens junctions were hyperphosphorylated in G12D-mutant cells. On the other hand, G13D mutations resulted in the hyperphosphorylation of MAPK family members (MAPK7, MAPK9, MAPK10) and proteins involved in GTP/GDP binding, RNA translation, and protein folding [24]. Both studies consistently identified ALDH3A1 as upregulated in KRAS^G13D^ cells compared to KRAS^G12D^ cells, highlighting a potential biomarker for codon-specific KRAS signaling in CRC.

Although both KRAS^G12X^ mutations and the BRAF V600E mutation are key drivers of cell proliferation and survival in CRC through the activation of the MAPK (RAS/RAF/MEK/ERK) signaling pathway, they are associated with distinct clinical outcomes [47]. To investigate the molecular basis underlying the divergent effects of KRAS and BRAF mutations, Kundu et al. employed an integrative multi-omic approach, including transcriptomics, proteomics, and metabolomics, to investigate various isogenic KRAS or BRAF V600E mutations in RKO CRC cell lines. Their analysis aimed to identify differentially regulated pathways associated with specific KRAS mutations (G12D and G13D) and the BRAF V600E mutation [48]. Global proteomic profiling revealed that the G12D mutation was associated with the enrichment of pathways involved in BRCA1-mediated DNA damage response, mismatch repair, and cell-cycle checkpoint regulation compared to Ras-pathway WT cells. In contrast, KRAS^G13D^-mutant cells showed enrichment in pathways related to actin-based motility, Rho GTPase signaling, and axonal guidance, suggesting distinct roles in cytoskeletal dynamics and cellular migration [48]. Canonical pathway analysis was performed on differentially expressed proteins to compare how KRAS^G12D^ and KRAS^G13D^ mutations alter signaling relative to CRC cells harboring the BRAF V600E mutation. The results revealed that KRAS^G13D^-mutant cells exhibited upregulation of the Wnt/β-catenin signaling pathway, whereas KRAS^G12D^-mutant cells showed enrichment in the serine biosynthesis pathway compared to BRAF V600E-mutant RKO cells [48].

**Figure 2:**
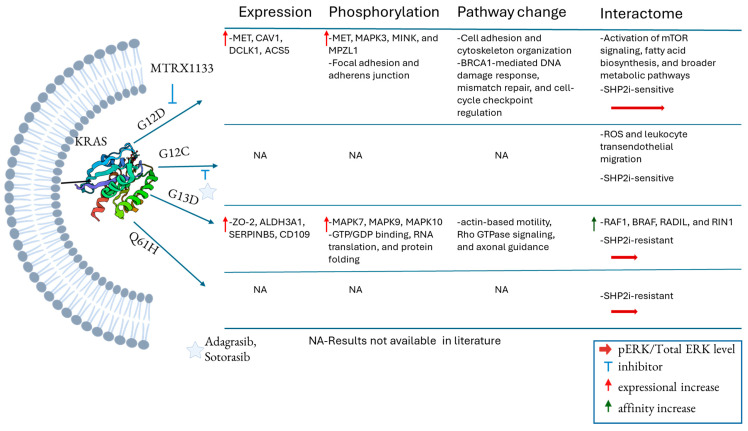
Differential signaling pathways and functional networks associated with various KRAS mutations, as revealed by current studies. The table is summarized from references [23,32, 33,36,38].

The observation of unique KRAS signaling associated with individual KRAS mutants is further supported by the findings that WT and KRAS^G12V/D^ are sensitive to SHP2 inhibitors, while KRAS^G13D^ and KRAS^Q61H^ are resistant to SHP2 [49], encoded by gene *PTPN11*, which dephosphorylates the tyrosine phosphorylation of KRAS at Y32 and Y64 [50]. Biolayer interferometry and mass spectrometry revealed that double phosphorylation of KRAS^Q61H^ reduces its binding to BRAF by 4-fold compared to its unphosphorylated form. For WT KRAS, phosphorylation weakens BRAF binding by 15-fold. Notably, the Q61H mutation reduces phosphorylation-dependent downregulation of KRAS activity, a feature not observed in other mutations like G12D/V [51]. While the functional changes driven by specific KRAS mutations remain to be fully elucidated, Figure 2 summarizes recent proteomic findings on KRAS mutation-associated alterations in the proteomic landscape, functional networks, and signaling pathways.

## 4. Differential Interactomes Specific to KRAS Mutations

Although the mechanisms underlying differential signaling driven by distinct KRAS mutations remain incompletely understood, it is hypothesized that these differences arise from altered GTPase activity or changes in effector binding affinity, potentially due to the introduction of bulky amino acid residues. To investigate altered signaling due to different KRAS mutations, immunoprecipitation followed by mass spectrometry-based proteomics was employed to characterize the differential interactomes of WT and mutant KRAS proteins expressed in HKe-3 CRC cells, derivatives of HCT116 cells in which the endogenous KRAS G13D allele has been knocked out [31]. The analysis revealed that each KRAS mutant exhibits unique binding affinities for downstream effectors within the RAS signaling pathway, including RAF1, BRAF, RADIL, RIN1, and MAPK3. Notably, the KRAS^G13D^ mutant demonstrated the highest affinity for RAF1, BRAF, RADIL, and RIN1 compared to both WT and KRAS^G12V^ in HKe-3 cells, while showing the lowest affinity for MAPK3. These findings support the notion that specific KRAS mutations can differentially modulate downstream signaling through selective effector engagement [31]. A similar investigation in HEK293 cells expressing KRAS variants, utilizing TurboID-based proximity labeling [52] followed by quantitative proteomics, revealed shared and mutation-specific alterations in KRAS-interacting proteins among KRAS variants. While canonical KRAS binding partners, including proteins in the MAPK and PI3K-AKT pathways, were commonly enriched across WT and mutant KRAS-expressing cells, distinct differences were observed among specific mutants. The interacting proteins from KRAS^G12V^ and KRAS^G12D^ mutants were associated with the enhanced activation of mTOR signaling, fatty acid biosynthesis, and broader metabolic pathways. In contrast, the interacting proteins from KRAS^G12C^-expressing cells showed enrichment in pathways related to chemical carcinogenesis involving reactive oxygen species (ROS) and leukocyte transendothelial migration [53]. The data highlight mutation allele-specific alterations in KRAS interactomes, which may partly account for the different oncogenic potential of KRAS variants. The differential interactomes specific to KRAS mutations revealed by proteomic studies are summarized in Figure 2.

## 5. KRAS Conformational Changes Induced by Mutations

Masson et al. employed hydrogen–deuterium exchange mass spectrometry (HDX-MS) to investigate the mutation-induced conformational changes in KRAS and the conformational changes resulting from its binding to other effector proteins [54]. HDX-MS analysis showed that the KRAS^V14I^ mutation, implicated in Noonan syndrome and lung cancer [55], introduced increased flexibility in P-loop residues (12–19) and destabilized the switch I region, as compared to WT KRAS. This conformational change exposes the β2/β3 strands to solvent, facilitating GDP release and enhancing the binding affinity for the guanine nucleotide exchange factor (GEF) SOS1 [56]. Consistent with the mutation-induced extended conformation, HDX-MS analysis of G12D and WT KRAS bound with GTP showed increased flexibility in residues 2–19 and 115–125 due to the KRAS^G12D^ mutation [57]. However, other structural studies have shown that an extended conformation of switch I can also occur in WT KRAS, attributed to conformational changes around residue A59 induced by the flexibility of the N-terminal region. This N-terminal flexibility is stabilized upon removal of the initiator methionine and subsequent N-terminal acetylation [58]. The binding of inhibitors to KRAS^G12D^ and KRAS^G12C^ decreased the deuterium uptake in the switch I region and P-loop of KRAS, which suggests that these regions are stabilized and perhaps shows a reduced interaction of GEFs of KRAS [57,59]. These findings collectively highlight that KRAS mutations induce distinct structural and functional alterations that reprogram signaling and metabolic pathways in an allele-specific manner, with important implications for targeted therapeutic strategies.

## 6. Post-Translational Modifications of KRAS and Their Involvement in Signaling

Mass spectrometry has proven to be a powerful tool for identifying novel PTMs and examining the PTM status of KRAS [60]. The most commonly identified KRAS PTMs are lipidation, phosphorylation, acetylation, ubiquitination, SUMOylation, and nitrosylation [61].

KRAS undergoes a series of PTMs that are essential for its localization and function. First, KRAS is farnesylated at cysteine 185 (C185) within the CAAX motif at C-terminal, where a 15-carbon farnesyl lipid is added. This is followed by the removal of the terminal AAX residues and the subsequent carboxymethylation of the newly exposed C-terminal cysteine [62]. Together with the lipidation at C185, the polybasic chain in the hypervariable region facilitates the stable anchoring of KRAS to the inner leaflet of the plasma membrane, which is critical for initiating downstream signaling pathways [63,64]. The anchoring of KRAS on the plasma membrane enables the dimerization of RAS, which is essential for the activation of Raf-1, a serine/threonine kinase in the MAPK pathway [65,66]. To further investigate the dimerization of KRAS and its effect on KRAS signaling, top-down proteomics on KRAS-GDP or KRAS-GTP reconstituted in a proteoliposome was used, and the results indicate that KRAS is dimerized on membranes in the presence of GTP [67]. The dimeric conformation depends on lipid composition and is disrupted by the binding of effector protein SOS1 [67].

Besides carboxyl methylation during CAAX motif processing, a “second signal” immediately upstream of the CAAX motif is necessary to further enhance its association with the plasma membrane. For KRAS isoform 4A, this second signal is a palmitoylation occurring on cysteine 180 at the C-terminus via thioester linkages and is referred to as S-palmitoylation. Upon palmitoylation, RAS acquires a 100-fold greater affinity for membranes than that of the prenylated-only protein [68].

KRAS has been shown to undergo methylation at Lys184 and Lys182. Through immunoaffinity purification followed by quantitative proteomics, the SET domain-containing histone lysine methyltransferase 7 (SETD7) was identified as the enzyme responsible for methylating KRAS at these sites. This methylation promotes the proteosome-dependent degradation of KRAS, mediated by the RABGEF1 E3 ligase [69]. As a result, the KRAS level decreases, leading to the attenuation of the KRAS/MEK/ERK signaling.

Phosphorylation is another PTM in KRAS that controls the signaling and translocation of KRAS from the plasma membrane to the endoplasmic reticulum. Top-down and bottom-up proteomics revealed that residues Tyr32 (switch I) and Tyr64 (switch II) were phosphorylated by Src kinase, and phosphorylation on KRAS at these sites altered the confirmation of the switch I and switch II regions, impairing the binding to effectors [50]. The biolayer interferometry analysis for the binding of phosphorylated KRAS with effector protein BRAF showed a decrease in the binding affinity of effector protein BRAF [50]. The other residue in KRAS that becomes phosphorylated is Ser181, catalyzed by protein kinase C (PKC), and is implicated in the dissociation of KRAS from the plasma membrane because of decreased electrostatic interaction between the polybasic chain and with negatively charged phospholipids, particularly phosphatidylserine [70].

Another key PTM in KRAS is acetylation, whose effect on the activity of KRAS depends on the location of the acetylated residue. Mass spectrometric analysis shows that KRAS is acetylated at residues Lys104, Lys147, and the N-terminus after removal of initiator methionine (iMet) [58]. Studies have shown that the acetylation of Lys147 is mediated by HDAC6 and deacetylated by SIRT2 [71]. An acetylated mimic of KRAS^K147Q^ showed higher nucleotide exchange kinetics as compared to KRAS WT or KRAS^G12V^, measured by nucleotide exchange of fluorescently labeled GDP and GTP, suggesting the acetylation of Lys147 accelerates the signaling due to favoring the active form of KRAS [72]. Functional assays measuring nucleotide exchange rates revealed that the KRAS^K147Q^ mutant does not accurately mimic the acylated lysine, and that acetylation at Lys147 does not significantly affect the rate of SOS-mediated nucleotide exchange [73]. The other acetylation site in KRAS is Lys104, which is modified by the acetyltransferase p300 and deacetylated by the NAD^+^-dependent deacetylase SIRT1 [74]. Although Western blot analysis of phosphorylated AKT and ERK showed decreased activity of the KRAS^G12C-K104R^ mutation compared to KRAS^G12C-K104^, suggesting that acetylation at Lys104 downregulates KRAS activity [74,75], studies by Lammers’s group reported that KRAS activity is unaffected by the acetylation status of Lys104, indicating that the glutamine substitution at lysine is a poor mimic of acetylation [73]. Other acetylation is at the N-terminus of KRAS. Mass spectrometric analysis of KRAS overexpressed in HEK293 cells suggested that KRAS undergoes PTM at the N-terminus, which involves the removal of iMet followed by the acetylation of the N-terminus, and this PTM is absent when KRAS is overexpressed in a prokaryotic system. This PTM is important for the stabilization of the binding site of Mg^2+^, as the N-acetyl group interacts with the central beta sheet and stabilizes the switch regions [58]. Although the effect of acetylation at residues Lys104 and Lys147 on regulating KRAS oncogenicity is inconsistent, it is unequivocally demonstrated that inhibition or knockdown of deacetylases such as SIRT1/2 and HDAC6 reduces the mutated KRAS-mediated tumor progression.

Proteomic analysis of ubiquitinome has suggested that KRAS undergoes ubiquitination at positions Lys104, Lys128, and Lys147, where ubiquitination at each position has different signaling outcomes. It was shown by the pulldown assay that monoubiquitination at Lys147 increases the KRAS downstream signaling by increasing the binding of GTP and other downstream effector proteins, including PI3K and Raf [76]. On the other hand, the ubiquitination-deficient mutant K128R KRAS showed higher levels of phosphorylated ERK1/2 and MEK1/2, suggesting ubiquitination at Lys128 decreased signaling because of increased binding of RAS GTPase-activating protein, such as NF1 and RASA1 [77]. Compared to Lys147 and Lys128, Lys104 is less frequently ubiquitinated and does not alter the GAP-mediated GTP hydrolysis, suggesting that only more prevalent ubiquitination (Lys147 and Lys128) leads to different downstream signaling modulation [78].

Immunoprecipitation followed by top-down proteomics identified nitrosylation as another PTM in KRAS using CRC cell lines DLD-1 and HCT116 [79]. In DLD-1 and HCT116 CRC lines, both WT and KRAS^G13D^ were present, and when the G13D mutant allele was removed while keeping the WT KRAS, the top-down proteomics identified the nitrosylation of KRAS at C118 in more than 90% of the WT KRAS protein [79]. Based on the observation of nitrosylation only for WT KRAS after removing G13D alleles in DLD-1 and HCT115 (KRAS WT/G13D), it was hypothesized that nitrosylation at C118 is the key KRAS activation pathway when the activating mutation (i.e., G13D) is absent. Nitrosylation at C118 (binding site of GDP) promotes the dissociation of GDP by electron transfer between C118 and GDP and activates KRAS, which is followed by increased downstream signaling [80]. The mutation of C118 of KRAS by C118D and C118S (oxidized mimic of C118) inhibited the nitrosylation-mediated KRAS activation [81,82]. The molecular dynamics simulation of KRAS^C12D/C118S^ showed an altered structure in nucleotide-binding regions and the switch regions [83]. Several other studies have suggested that the reaction of nitric oxide with C118 of WT KRAS promotes KRAS activation and exerts an oncogenic effect on KRAS-mediated tumor growth and proliferation [84]. The List of common PTMs of KRAS and their known implications in signaling are summarized in Table 2.

## 7. Current Progress in Inhibiting Different Mutations of KRAS

Since KRAS was identified as a key oncogene, attempts to block its activity by inhibiting GTP binding have mostly failed. This is due to the lack of a clear binding site for inhibitors and KRAS’s extremely high affinity for GTP, which is abundant inside cells [85]. This prompted exploration of alternative targets, including upstream receptor tyrosine kinases and downstream effectors such as ERK and MEK [86,87]. However, targeting effector proteins in KRAS-mutant cancers proved ineffective, as resistance emerged through the activation of alternative pathways or additional mutations in those effectors [88].

Despite the inherent difficulties in targeting the “undruggable” KRAS protein, numerous efforts have been undertaken to identify small molecule inhibitors. Shokat and colleagues developed a KRAS^G12C^ inhibitor, compound 12, which contains an acrylamide group that irreversibly binds to the cysteine at position 12, forming a stable thioether adduct, thereby forming switch II regions [89]. Intact protein mass spectrometry confirmed the covalent binding of the inhibitor to KRAS^G12C^, while BSA, despite having a free cysteine, remained unmodified [89]. Inspired by this work, ARS 1620 was developed with better inhibitory activity and bioavailability for KRAS^G12C^ mutant cancer [90]. An unbiased chemical proteomic screen revealed that the KRAS^12C^ was chemically modified, with off-target modification at FAM213A^85C^ and AHR^639C^. Although the effect of off-target modification of ARS1620 remains to be explored, the dose-dependent reduction in patient-derived tumor volume with only the KRAS^G12C^ mutation on the administration of ARS 1620 showed its potential in inhibiting tumors with KRAS^G12C^ mutation [90]. Incorporating the quinazolinone core from ARS 1620 and several modifications on their starting compound, Amgen developed the first FDA-approved drug AMG510 (sotorasib) to treat NSCLC with KRAS^G12C^ mutations. Cysteine proteome analysis showed the selective modification of KRAS^G12C^ with no off-target activities of AMG510. A clinical trial of AMG510 with 4 patients with NSCLC showed objective partial response with decreased tumor size for two patients and stable disease in the other two patients [91]. The other FDA-approved KRAS^G12C^ inhibitor, MTRX849 (adagrasib) for treating NSCLC, was developed through the collaborating efforts of Mirati Therapeutics and Array BioPharma by incorporating a series of modifications on a tetrahydropyridopyrimidine scaffold [92]. Targeted proteomic analysis monitoring the peptides containing Cys-12 of KRAS from tissue samples showed similar overall potency of MTRX849 (35 ± 0.3 mM^–1^ s^–1^) compared to ARS-1620 (1.1 mM^–1^ s^–1^) and AMG 510 (9.9 mM^–1^ s^–1^), with high selectivity for KRAS^G12C^, as confirmed by a cysteine profiling assay performed by mass spectrometry in NCI-H358 cells [93].

Divarasib is another highly potent inhibitor for targeting KRAS^G12C^ mutation-containing cancer. Studies across multiple cell lines suggest that divarasib is 16,000 times more selective for the KRAS^G12C^ mutation compared to non-G12C mutated cell lines. A cell viability assay in preclinical studies employing MIA PaCa-2 cells with KRAS^G12C^ mutation showed that divarasib (IC_50_: 0.19 nM/L) was >50-fold more potent than sotorasib (IC_50_: 12.75 nM/L) and adagrasib (IC_50_: 17.88 nM/L) [94], and it was 50 times more selective than sotorasib and adagrasib [95,96]. In a Phase I/II clinical trial for NSCLC, divarasib achieved an overall response rate (ORR) of 53.4% and a progression-free survival (PFS) of 13.1 months. As a comparison, sotorasib showed an ORR of 14% and a PFS of 6.3 months [97]. Divarasib is currently being evaluated in combination with pembrolizumab in a Phase III trial (KRYSTAL-2) for NSCLC [98].

The KRAS^G12D^ mutation is the most prevalent mutation in CRC and PDAC, and patients with this mutation have the worst prognosis compared to other KRAS mutations. Unlike G12C mutations, KRAS^G12D^ lacks a reactive group for covalent inhibitors, making it unsuitable for covalent inhibition. MTRX1133 is the first non-covalent inhibitor developed specifically for KRAS^G12D^, and it demonstrated picomolar affinity and approximately 700-fold selectivity over WT KRAS [99]. Unlike covalent inhibitors such as adagrasib and sotorasib, which block KRAS activation by preventing GTP loading, the binding of MTRX1133 induces a conformational change in the switch II region, disrupting critical protein–protein interactions necessary for downstream KRAS signaling [22].

KRAS^G12S^ accounts for 4.4% of all KRAS mutations. To target this variant, a β-lactone electrophile was added to the tetrahydropyridopyrimidine core of MRTX849, resulting in the inhibitor G12Si-1. This compound forms a covalent ester bond through nucleophilic attack by the serine hydroxyl group at position 12. Targeted analysis of KRAS^G12S^ showed that G12Si-1 covalently modified the G12S of KRAS. The optimized version of this inhibitor, G12Si-5, achieved complete modification of KRAS^G12S^ within 10 min, as measured by targeted proteomics. Using competition proteomics, a proteomic profiling after treatment with a terminal alkyne-containing analog of G12Si-5 in cells that were pretreated with G12Si-5 or DMSO, resulted in KRAS^G12S^ being a selective target together with off-target modification on proteins PLD3, TRMT61A, and TRMT6 [100].

In PDAC, the KRAS^G12R^ accounts for 17% of all KRAS mutations [101]. To target this variant, the Shokat group developed an inhibitor containing an α,β-diketoamide reactive group, which forms an imidazolium adduct with KRAS^G12R^, as confirmed by crystal structure and proteomic analysis [102]. Although this compound selectively modifies KRAS^G12R^ in cell lysate, it could not modify endogenous KRAS in BaF3 murine cells expressing KRAS^G12R^ at concentrations below 100 μM [103]. Lack of endogenous activity was attributed to the fact that the majority of KRAS in cells were GTP-bound KRAS^G12R^, and experiments showed that the inhibitor is only active in GDP-bound KRAS [103].

Lito and colleagues developed a pan-KRAS inhibitor by removing the reactive group of the KRAS^G12C^ inhibitor BI-0474 and modifying the resulting compound to create BI-2865 by structure-based drug design. This inhibitor targets both WT and a range of KRAS mutants with an IC_50_ of 5–140 nM [104]. BI-2865 inhibitory activity in cells was primarily due to targeting GDP-bound KRAS, and the binding of the inhibitor reduced the SOS1-promoted nucleotide exchange required for KRAS activation [104]. Other studies showed that pan-KRAS inhibitors BI-2865 and BI-2493 are particularly effective for treating gastroesophageal cancers, in which WT KRAS is amplified with a copy number greater than 7. Both pan-KRAS inhibitors prevent nucleotide exchange, locking KRAS in the inactive state [105].

A different approach to treat KRAS-mutated cancer cells is by treating cells with a small bifunctional molecule, a proteolysis targeting chimeras (PROTACs), which engages the E3 ubiquitin ligase to KRAS to ubiquitinate KRAS for proteasomal degradation [106]. LC-2 was the first PROTAC developed to degrade KRAS^G12C^ by recruiting VHL or cereblon E3 ligase and is covalently linked to MTRX849, a KRAS^G12C^ inhibitor [107]. However, LC-2 engages the already inhibited KRAS^G12C^ by covalent modification, and it lacks the catalytic mode of action, requiring a 1:1 stoichiometric relationship between PROTAC and KRAS. Popow et al. developed the first non-covalent pan-KRAS PROTAC, ACBI3, that can degrade 13 out of 17 KRAS mutants, sparing WT KRAS [108]. Cryo-EM structure studies and whole-cell proteomic studies indicated that ACBI3 assumes a fishhook structure and engages the VHL E3 ligase with KRAS for ubiquitination, and the KRAS protein is selectively degraded, sparing HRAS and NRAS [108]. Besides this, the use of small interfering RNA for silencing KRAS RNA, the use of glue molecules that facilitate the complex formation of KRAS with the non-canonical binding partner cyclophilin A (CYPA), and the use of peptide nucleic acids (PNAs) are other approaches that are under investigation for inhibiting oncogenic KRAS [109]. A combination of a KRAS inhibitor with an immune checkpoint inhibitor (ICI) is being investigated in clinical trial for treating cancers that are refractory to ICI-based therapy [110].

## 8. Resistance Mechanisms in Targeted Inhibition of KRAS

Although sotorasib and adagrasib have been approved for treating NSCLC, the ORR is 37–43% and the median PFS is 6–7 months [111]. The modest outcomes are attributed to both primary and acquired resistance mechanisms when targeting different oncogenic KRAS alleles [112]. Understanding these resistance mechanisms is essential for improving KRAS-targeted therapies or developing effective combination strategies.

From an unbiased enrichment analysis, the primary resistance has been linked to loss-of-function mutations in tumor suppressor genes such as *KEAP1*, *SMARCA4*, and *CDKN2A*, which are frequently observed and correlated with poor PFS in patients treated with a KRAS^G12C^ inhibitor, sotorasib or adagrasib [113]. In addition, this analysis identified the amplification of *KRAS*, co-mutation of gene encoding PI3K/AKT/MTOR pathways, and co-mutation of other oncogenes such as *ALK*, *ROS1*, and *NTRK3* as primary resistance mechanisms in KRAS inhibitors [113].

Acquired resistance to adagrasib has been identified in NSCLC, CRC, and appendiceal cancers through the next-generation sequencing of tissues derived from patients [114]. This study showed the heterogeneous nature of acquired resistance mechanisms, where mutations of residues Y96C, H95Q, H95R, and R68S from the adagrasib binding pocket of KRAS were observed. In addition, activating mutations G12D/V/R, G13D, and Q61H in KRAS were identified as an acquired resistance [114]. Additional mutations in *EGFR*, *BRAF*, *MAP2K1*, and *MEK* were observed, indicating that the inhibition of KRAS can be bypassed by activating mutations in the upstream or downstream RAS pathway [114].

In PDAC patients with *KRAS*^G12C^ mutation, circulating tumor DNA sequencing revealed primary resistance mechanisms, including the high-level amplification of *MYC*, *KRAS^G12C^*, *BRAF*, *ERBB2*, *CDK4*, and increased variant allele frequency of a pre-existing subclonal KRAS^G12R^ [115]. As acquired resistances in PDAC after treatment with a G12C inhibitor, the amplification of *KRAS^G12C^*, amplification of *EGFR*, activating *PIK3CA* mutations, and oncogenic *NFE2L* mutations were identified. A case with a *KRAS^A146P^* mutation was identified as acquired resistance in the KRAS^G12C^ inhibitor; no additional mutations in KRAS were identified as acquired resistance in PDAC, contrasting with NSCLC and CRC acquired resistance [115].

For KRAS^G12D^, the primary resistance to MRTX1133 was studied using copy number analysis, bulk RNA sequencing, and RPPA. Results indicated that the RTK-RAS pathway and associated genes (*EGFR*, *MET*, *BRAF*, *ETV1*) and EMT regulator genes (*ZEB1*, *TWIST*) were upregulated [115]. The RPPA analysis showed the upregulation of proteins involved in the PI3K-AKT-mTOR pathway (p85α, p110α, mTOR, Rictor, pS6), translation regulators (eIF4G, eEF2K), and increased expression of BRAF and phosphorylated BRAF [115].

To identify the acquired resistance of MTRX1133, resistant cell lines were developed by escalating concentrations of MTRX1133, and whole-exome sequencing was performed. Although this study did not identify the acquired additional point mutation in *KRAS* or downstream Ras pathways, the high amplification of the copy number of gene *CDK6*, *CDK8*, and *ABCB1A/B* was observed, consistent with resistance mechanisms seen in PDAC treated with adagrasib [115]. This result indicates that resistance to KRAS treatment is developed through the dysregulation of the cell cycle by CDK6 and the efflux of drugs using the ABCB1A/B mechanism.

Chen and colleagues examined acquired resistance to KRAS inhibition in pancreatic cancer cell lines from both mice and patients [116]. Their study found that resistant cells activated the unfolded protein response (UPR) via IRE1α to counteract disruptions in protein homeostasis caused by KRAS inhibition [116]. IRE1α splices a 26-nucleotide segment and deletes a frame shift mutation from unspliced *XBP1* mRNA, producing a mature *XBP1* mRNA that encodes a transcription factor that drives the expression of genes involved in the biogenesis of the protein secretory pathway, protein folding and secretion, and the removal of misfolded proteins from the endoplasmic reticulum [117]. In resistant cells, IRE1α was phosphorylated at S525, S529, S549, and T973 by AKT and ERK, which prevented its degradation by inhibiting SEL1L/HRD1-mediated ubiquitination [116]. Upregulation of multiple RTKs, as shown by RPPA and RNA sequencing, supports the role of IRE1α in maintaining protein homeostasis and contributing to resistance. Collectively, Table 3 summarizes the current resistance mechanisms to KRAS inhibitors.

## 9. Conclusions and Future Perspectives

KRAS is one of the most frequently mutated oncogenes implicated in multiple cancers. Because KRAS lacks an accessible drug-binding pocket and because of its picomolar affinity for GTP, designing inhibitors targeting nucleotide exchange pathways has long been difficult. However, with the recent FDA approval of drugs targeting the KRAS^G12C^ mutant, there is growing research interest in developing inhibitors for other allelic mutants. Despite these advances, both primary and acquired resistance to KRAS inhibitors remain significant obstacles in the effective treatment of cancer. Additionally, tumor heterogeneity in solid tumors such as PDAC contributes to reduced drug efficacy and complicates therapeutic outcomes. As pan-KRAS inhibitors and pan-KRAS PROTACs emerge as new treatment strategies with encouraging preclinical efficacy, extensive proteomic studies are needed to evaluate not only target engagement and degradation but also off-target effects, compensatory signaling, and the suppression of downstream pathways. Another critical area of investigation is the tumor microenvironment. Multi-omic analyses have revealed that KRAS-mutated CRC tumors can be classified into two distinct subtypes, KM1 and KM2. KM2 tumors exhibit upregulation of EMT, TGF-β signaling, and angiogenesis, and are associated with worse prognosis and increased stromal invasion [23]. Research has also shown that KRAS inhibition can shift cell states toward increased oxidative stress and enhanced extracellular matrix remodeling [118]. Therefore, future studies that focus on characterizing both tumor and stromal/immune proteomes at single-cell resolution or through spatial proteomics could provide novel insights to guide combination therapies that target both cancer cells and their surrounding microenvironment.

These advances present new challenges for proteomics in comprehensively characterizing the molecular and functional changes underlying these mechanisms. As essential functional biomolecules, proteins and their modifications play critical roles in virtually all diseases and drug resistance. Proteomic and mass spectrometric approaches, particularly those focused on interactomes, single-cell analysis, PTMs, and proteome homeostasis, are increasingly vital for elucidating functional networks, resistance mechanisms, and tumor heterogeneity in the context of KRAS-targeted cancer therapies.

## Figures and Tables

**Table 1:** Proteomic Alterations in Oncogenic KRAS Mutations.

Category	Key Alterations	References
Global (Phospho)Proteomic Signatures	Distinct proteomic/phosphoproteomic subtypes	[30]
	↑ Feedback regulators (DUSPs, SPRYs)	
	↑ MAPK and PI3K phosphorylation	[24]
	↑ Mutation-specific interactome changes	[31]
Metabolic Reprogramming	↑ Glycolysis (HK2, PKM2, LDHA) and glucose transporter level	[32]
	↑ Glutamine metabolism (GLS)	
	↑ Altered lipid synthesis (FASN, CPT1A)	[3,21]
	↑ Redox/antioxidant proteins (NRF2 targets)	[33]
Cell Cycle and Proliferation	↑ Cyclins (D1, E2) and CDKs (CDK4/6, and CDK2)	[34]
	↓ Inhibitors p21 (CDKN1A), p27 (CDKN1B)	[35]
	↑ DNA replication proteins (MCMs, PCNA, DNA polymerases, replication origin licensing factors)	[36]
	↑ Mitotic regulators (Aurora, PLK1) and checkpoint kinases (Chk1/2)	[37]
Stress Response and Proteostasis	↑ Chaperones (HSP70, HSP90, HSP27)	[38]
	↑ Proteasome activity	[39]
	↑ Autophagy (LC3, ATG)	[40]
	↑ UPR (BiP, CHOP)	[41]
Tumor Microenvironment	↑ Cytokines (IL-6, TGF-β)	[42]
	↑ ECM remodeling (MMPs, integrins)	[4]
	↑ Exosome-mediated signaling	[43]
	↑ Stromal interactions alter proteome	[44]

↑ upregulation ↓ downregulation.

**Table 2:** Annotated post-translational modification of KRAS and their known roles in signaling.

PTM on KRAS	Residues	Implication in Signaling	References
Farnesylation	C185	Anchoring to PM and activation	[68]
methylation	K182 andK184	Downregulation of KRAS	[69]
Phosphorylation	Y32, Y64	Downregulation of KRAS	[50]
	S181	Dissociation from PM	[70]
Acetylation	K104 and K147	Inconclusive	[73,74,75]
Ubiquitination	K104	No effect	[78]
	K128	Increased signaling	[77]
	K147	Increased signaling	[76]
Nitrosylation	C118	Increased signaling if the mutant KRAS allele is depleted	[79]

**Table 3:** Resistant mechanisms in KRAS inhibition, as revealed by current studies.

KRAS Mutant	Tumor Region	Primary Resistance	Acquired Resistance	References
G12C	NSCLC, CRC, and appendiceal cancer	▪ Mutation in tumor suppressor genes *KEAP1*, *SMARCA4*, and *CDKN2A* ▪ Amplification of KRAS^G12C^ ▪ Co-mutation in oncogenes *ALK*, *ROS1*, and *NTRK3*	▪ Mutation in KRAS Y96C, H95Q, H95R, and R68S ▪ Mutation in BRAF, MAP2K1, and MEK	[113,114]
G12C	PDAC	▪ Amplification of *MYC*, *KRAS^G12C^*, *BRAF*, *ERBB2*, *CDK4*	▪ KRAS^A146P^ mutation ▪ Amplification of *EGFR* ▪ Mutation on *PIK3CA* and *NFE2L*	[115]
G12D	PDAC	▪ Amplification of *EGFR*, *MET*, *BRAF*, *ETV1*, and *EMT* regulators *ZEB1* and *TWIST*	▪ Amplification of genes *CDK6*, *CDK8*, and *ABCB1A/B*	[115]

## Data Availability

No new data were created or analyzed in this study. Data sharing is not applicable to this article.
